# Supplementary figures and images for: Spatial structure arising from neighbour-dependent bias in collective cell movement
Source: PeerJ. 2016 Feb 15;4:e1689. doi: 10.7717/peerj.1689 (PMC4756733; doi:10.7717/peerj.1689)

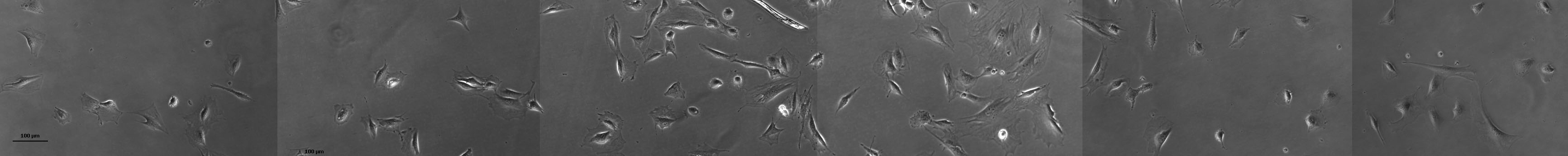

Supplement: Supplemental Information 1 — Time-lapse images of moving 3T3 fibroblast cells were captured, over a period of 12 h at 3 h intervals, using a light microscope and Eclipse TIS software at 100× magnification. Cell locations in each image were manually determined by superimposing markers onto cells and recording the Cartesian coordinates of markers using ImageJ image analysis software. [file peerj-04-1689-s003.zip › Supplemental_data/SupplementalFigS10.jpg]

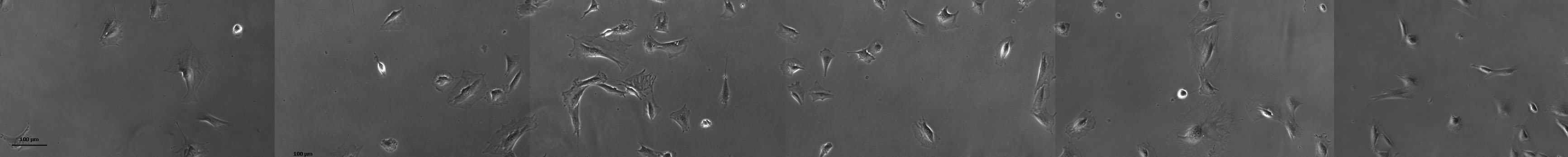

Supplement: Supplemental Information 1 — Time-lapse images of moving 3T3 fibroblast cells were captured, over a period of 12 h at 3 h intervals, using a light microscope and Eclipse TIS software at 100× magnification. Cell locations in each image were manually determined by superimposing markers onto cells and recording the Cartesian coordinates of markers using ImageJ image analysis software. [file peerj-04-1689-s003.zip › Supplemental_data/SupplementalFigS11.jpg]

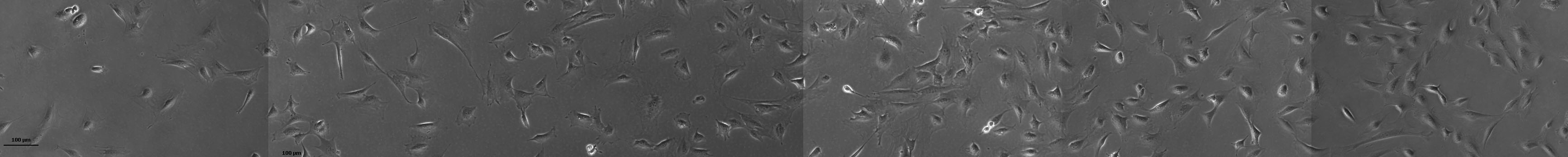

Supplement: Supplemental Information 1 — Time-lapse images of moving 3T3 fibroblast cells were captured, over a period of 12 h at 3 h intervals, using a light microscope and Eclipse TIS software at 100× magnification. Cell locations in each image were manually determined by superimposing markers onto cells and recording the Cartesian coordinates of markers using ImageJ image analysis software. [file peerj-04-1689-s003.zip › Supplemental_data/SupplementalFigS12.jpg]

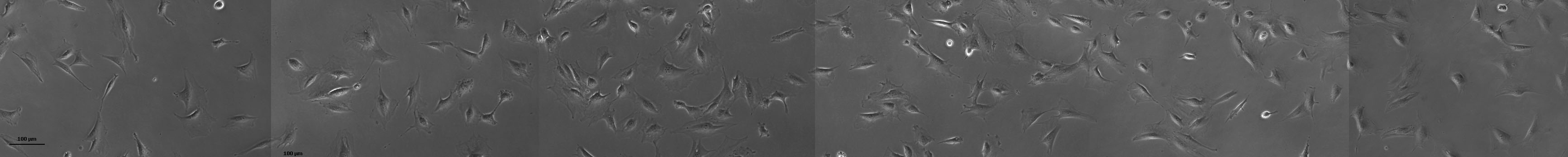

Supplement: Supplemental Information 1 — Time-lapse images of moving 3T3 fibroblast cells were captured, over a period of 12 h at 3 h intervals, using a light microscope and Eclipse TIS software at 100× magnification. Cell locations in each image were manually determined by superimposing markers onto cells and recording the Cartesian coordinates of markers using ImageJ image analysis software. [file peerj-04-1689-s003.zip › Supplemental_data/SupplementalFigS13.jpg]

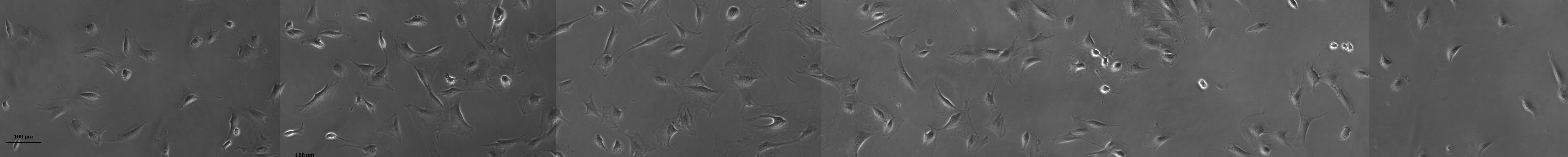

Supplement: Supplemental Information 1 — Time-lapse images of moving 3T3 fibroblast cells were captured, over a period of 12 h at 3 h intervals, using a light microscope and Eclipse TIS software at 100× magnification. Cell locations in each image were manually determined by superimposing markers onto cells and recording the Cartesian coordinates of markers using ImageJ image analysis software. [file peerj-04-1689-s003.zip › Supplemental_data/SupplementalFigS14.jpg]

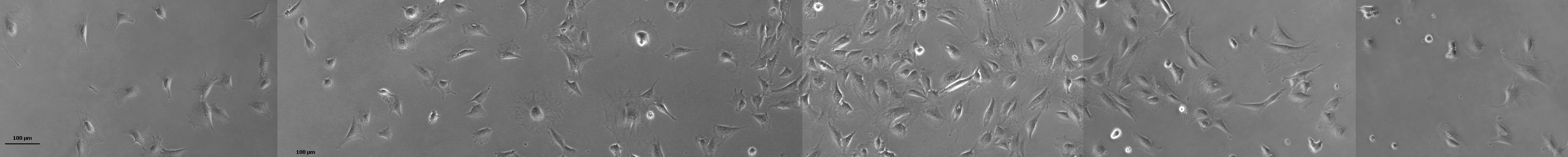

Supplement: Supplemental Information 1 — Time-lapse images of moving 3T3 fibroblast cells were captured, over a period of 12 h at 3 h intervals, using a light microscope and Eclipse TIS software at 100× magnification. Cell locations in each image were manually determined by superimposing markers onto cells and recording the Cartesian coordinates of markers using ImageJ image analysis software. [file peerj-04-1689-s003.zip › Supplemental_data/SupplementalFigS15.jpg]

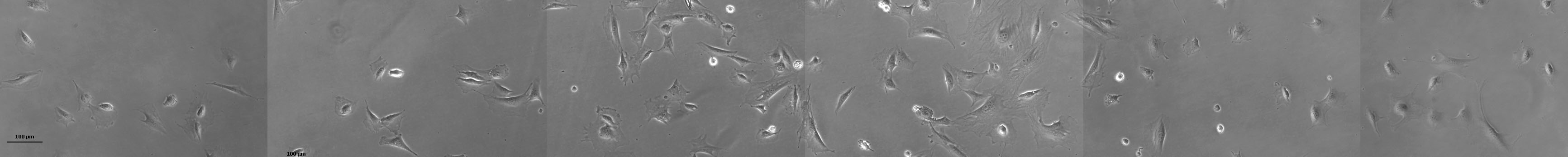

Supplement: Supplemental Information 1 — Time-lapse images of moving 3T3 fibroblast cells were captured, over a period of 12 h at 3 h intervals, using a light microscope and Eclipse TIS software at 100× magnification. Cell locations in each image were manually determined by superimposing markers onto cells and recording the Cartesian coordinates of markers using ImageJ image analysis software. [file peerj-04-1689-s003.zip › Supplemental_data/SupplementalFigS16.jpg]

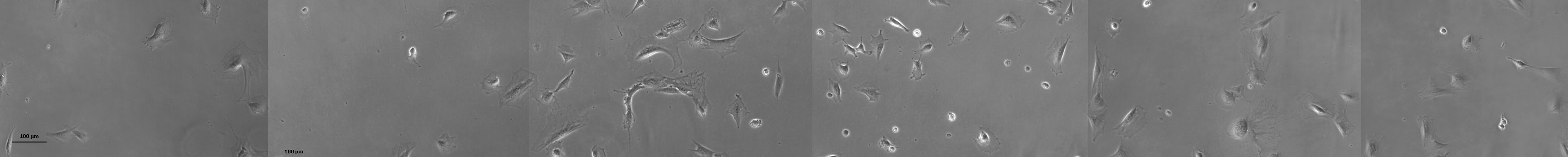

Supplement: Supplemental Information 1 — Time-lapse images of moving 3T3 fibroblast cells were captured, over a period of 12 h at 3 h intervals, using a light microscope and Eclipse TIS software at 100× magnification. Cell locations in each image were manually determined by superimposing markers onto cells and recording the Cartesian coordinates of markers using ImageJ image analysis software. [file peerj-04-1689-s003.zip › Supplemental_data/SupplementalFigS17.jpg]

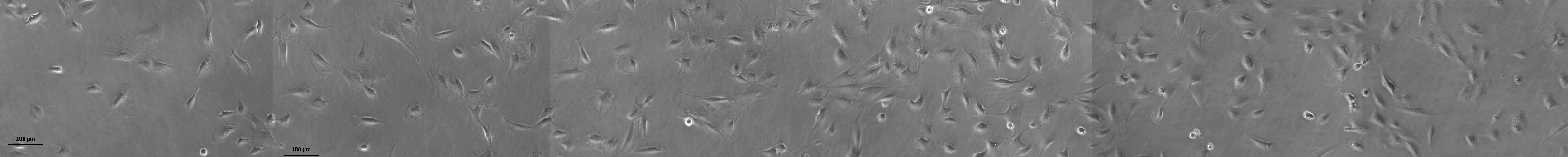

Supplement: Supplemental Information 1 — Time-lapse images of moving 3T3 fibroblast cells were captured, over a period of 12 h at 3 h intervals, using a light microscope and Eclipse TIS software at 100× magnification. Cell locations in each image were manually determined by superimposing markers onto cells and recording the Cartesian coordinates of markers using ImageJ image analysis software. [file peerj-04-1689-s003.zip › Supplemental_data/SupplementalFigS18.jpg]

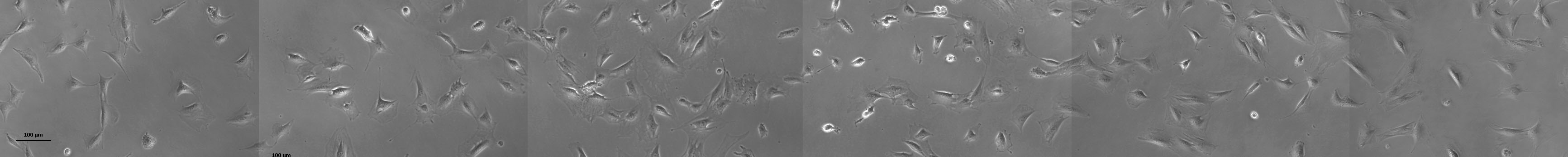

Supplement: Supplemental Information 1 — Time-lapse images of moving 3T3 fibroblast cells were captured, over a period of 12 h at 3 h intervals, using a light microscope and Eclipse TIS software at 100× magnification. Cell locations in each image were manually determined by superimposing markers onto cells and recording the Cartesian coordinates of markers using ImageJ image analysis software. [file peerj-04-1689-s003.zip › Supplemental_data/SupplementalFigS19.jpg]

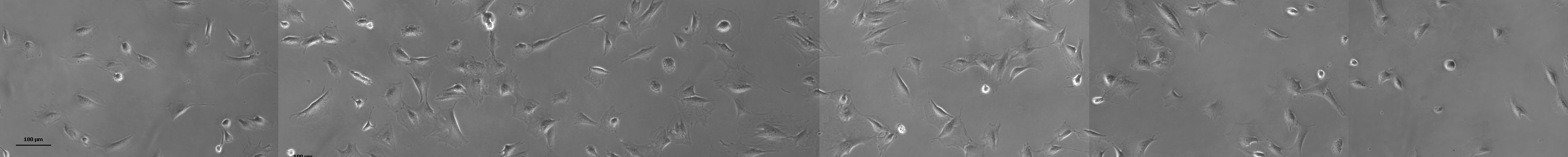

Supplement: Supplemental Information 1 — Time-lapse images of moving 3T3 fibroblast cells were captured, over a period of 12 h at 3 h intervals, using a light microscope and Eclipse TIS software at 100× magnification. Cell locations in each image were manually determined by superimposing markers onto cells and recording the Cartesian coordinates of markers using ImageJ image analysis software. [file peerj-04-1689-s003.zip › Supplemental_data/SupplementalFigS20.jpg]

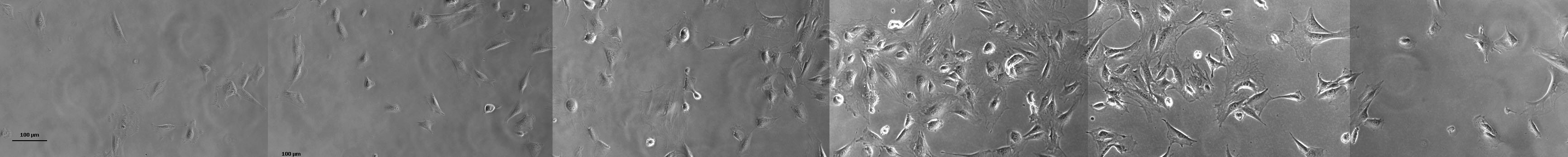

Supplement: Supplemental Information 1 — Time-lapse images of moving 3T3 fibroblast cells were captured, over a period of 12 h at 3 h intervals, using a light microscope and Eclipse TIS software at 100× magnification. Cell locations in each image were manually determined by superimposing markers onto cells and recording the Cartesian coordinates of markers using ImageJ image analysis software. [file peerj-04-1689-s003.zip › Supplemental_data/SupplementalFigS21.jpg]

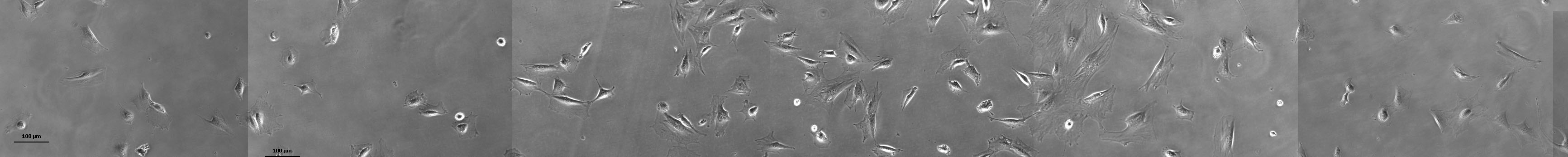

Supplement: Supplemental Information 1 — Time-lapse images of moving 3T3 fibroblast cells were captured, over a period of 12 h at 3 h intervals, using a light microscope and Eclipse TIS software at 100× magnification. Cell locations in each image were manually determined by superimposing markers onto cells and recording the Cartesian coordinates of markers using ImageJ image analysis software. [file peerj-04-1689-s003.zip › Supplemental_data/SupplementalFigS22.jpg]

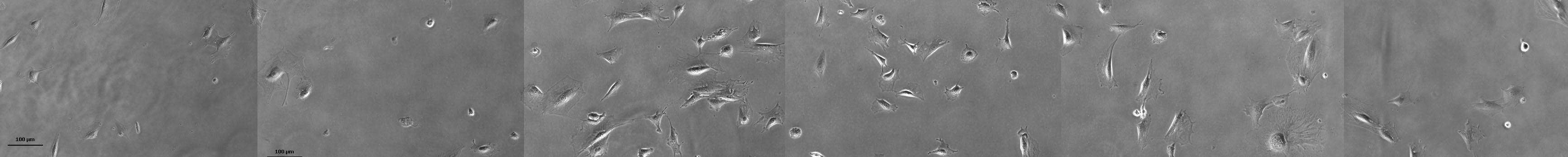

Supplement: Supplemental Information 1 — Time-lapse images of moving 3T3 fibroblast cells were captured, over a period of 12 h at 3 h intervals, using a light microscope and Eclipse TIS software at 100× magnification. Cell locations in each image were manually determined by superimposing markers onto cells and recording the Cartesian coordinates of markers using ImageJ image analysis software. [file peerj-04-1689-s003.zip › Supplemental_data/SupplementalFigS23.jpg]

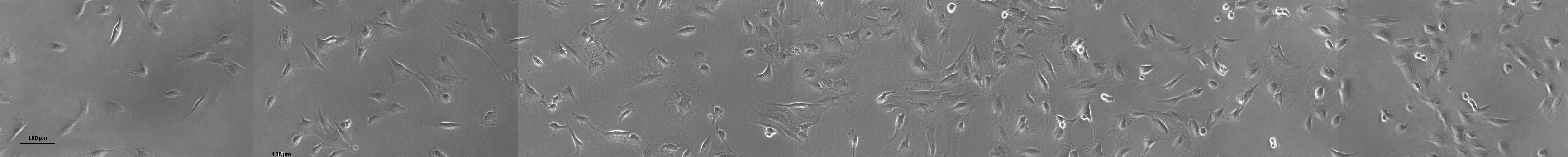

Supplement: Supplemental Information 1 — Time-lapse images of moving 3T3 fibroblast cells were captured, over a period of 12 h at 3 h intervals, using a light microscope and Eclipse TIS software at 100× magnification. Cell locations in each image were manually determined by superimposing markers onto cells and recording the Cartesian coordinates of markers using ImageJ image analysis software. [file peerj-04-1689-s003.zip › Supplemental_data/SupplementalFigS24.jpg]

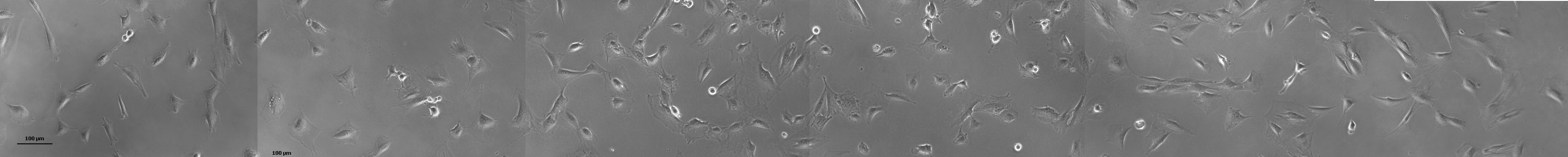

Supplement: Supplemental Information 1 — Time-lapse images of moving 3T3 fibroblast cells were captured, over a period of 12 h at 3 h intervals, using a light microscope and Eclipse TIS software at 100× magnification. Cell locations in each image were manually determined by superimposing markers onto cells and recording the Cartesian coordinates of markers using ImageJ image analysis software. [file peerj-04-1689-s003.zip › Supplemental_data/SupplementalFigS25.jpg]

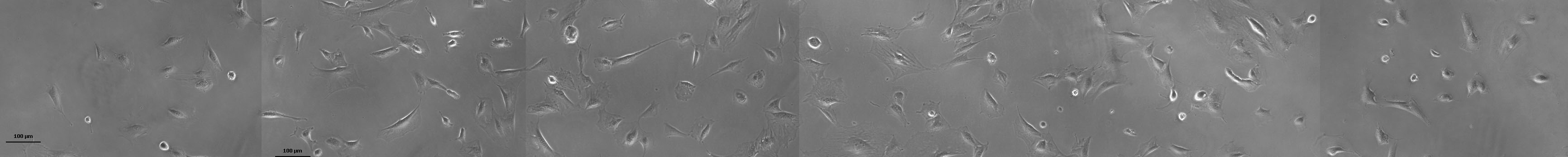

Supplement: Supplemental Information 1 — Time-lapse images of moving 3T3 fibroblast cells were captured, over a period of 12 h at 3 h intervals, using a light microscope and Eclipse TIS software at 100× magnification. Cell locations in each image were manually determined by superimposing markers onto cells and recording the Cartesian coordinates of markers using ImageJ image analysis software. [file peerj-04-1689-s003.zip › Supplemental_data/SupplementalFigS26.jpg]

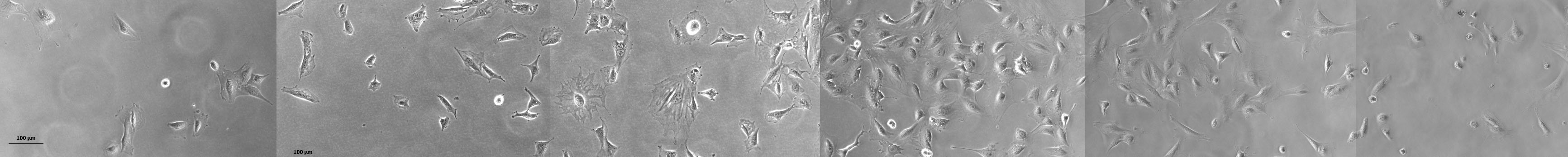

Supplement: Supplemental Information 1 — Time-lapse images of moving 3T3 fibroblast cells were captured, over a period of 12 h at 3 h intervals, using a light microscope and Eclipse TIS software at 100× magnification. Cell locations in each image were manually determined by superimposing markers onto cells and recording the Cartesian coordinates of markers using ImageJ image analysis software. [file peerj-04-1689-s003.zip › Supplemental_data/SupplementalFigS27.jpg]

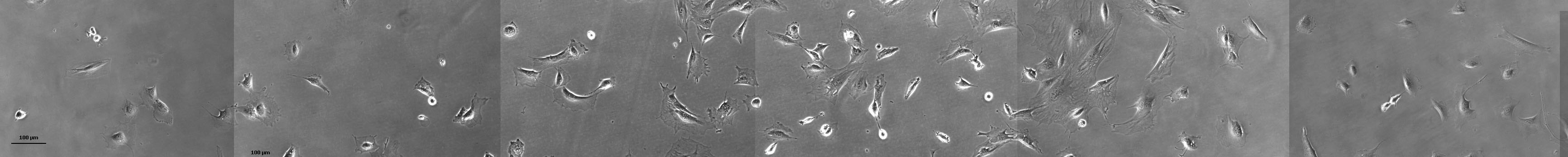

Supplement: Supplemental Information 1 — Time-lapse images of moving 3T3 fibroblast cells were captured, over a period of 12 h at 3 h intervals, using a light microscope and Eclipse TIS software at 100× magnification. Cell locations in each image were manually determined by superimposing markers onto cells and recording the Cartesian coordinates of markers using ImageJ image analysis software. [file peerj-04-1689-s003.zip › Supplemental_data/SupplementalFigS28.jpg]

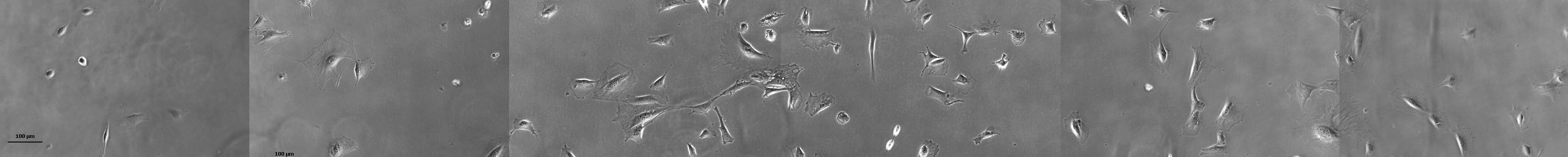

Supplement: Supplemental Information 1 — Time-lapse images of moving 3T3 fibroblast cells were captured, over a period of 12 h at 3 h intervals, using a light microscope and Eclipse TIS software at 100× magnification. Cell locations in each image were manually determined by superimposing markers onto cells and recording the Cartesian coordinates of markers using ImageJ image analysis software. [file peerj-04-1689-s003.zip › Supplemental_data/SupplementalFigS29.jpg]

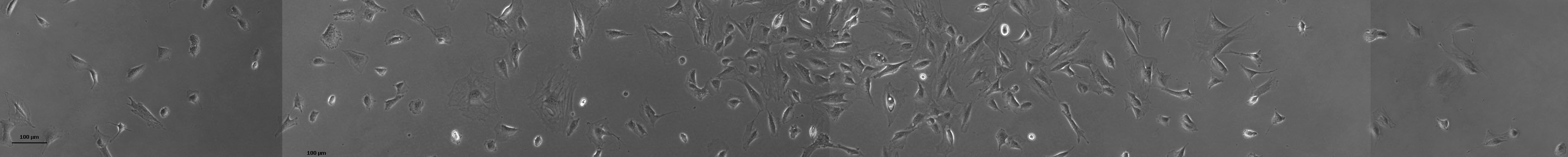

Supplement: Supplemental Information 1 — Time-lapse images of moving 3T3 fibroblast cells were captured, over a period of 12 h at 3 h intervals, using a light microscope and Eclipse TIS software at 100× magnification. Cell locations in each image were manually determined by superimposing markers onto cells and recording the Cartesian coordinates of markers using ImageJ image analysis software. [file peerj-04-1689-s003.zip › Supplemental_data/SupplementalFigS3.jpg]

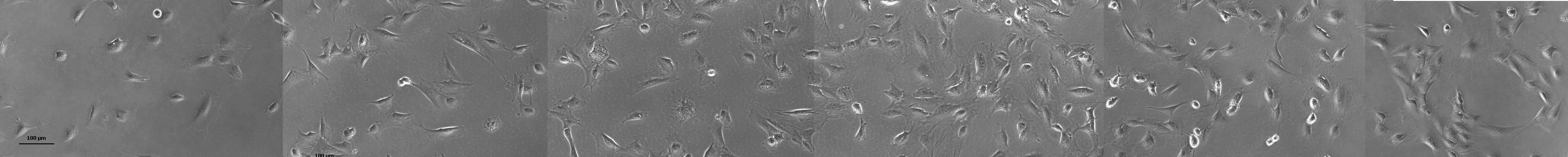

Supplement: Supplemental Information 1 — Time-lapse images of moving 3T3 fibroblast cells were captured, over a period of 12 h at 3 h intervals, using a light microscope and Eclipse TIS software at 100× magnification. Cell locations in each image were manually determined by superimposing markers onto cells and recording the Cartesian coordinates of markers using ImageJ image analysis software. [file peerj-04-1689-s003.zip › Supplemental_data/SupplementalFigS30.jpg]

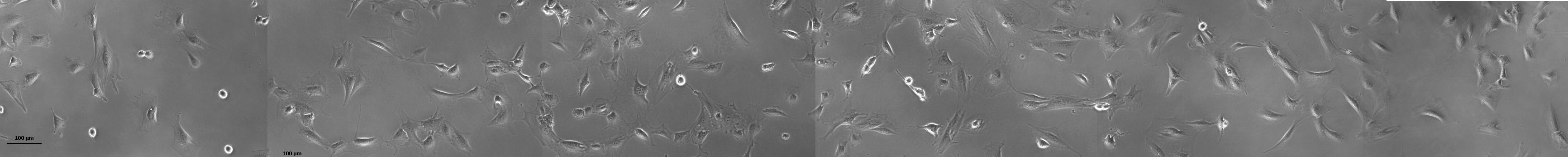

Supplement: Supplemental Information 1 — Time-lapse images of moving 3T3 fibroblast cells were captured, over a period of 12 h at 3 h intervals, using a light microscope and Eclipse TIS software at 100× magnification. Cell locations in each image were manually determined by superimposing markers onto cells and recording the Cartesian coordinates of markers using ImageJ image analysis software. [file peerj-04-1689-s003.zip › Supplemental_data/SupplementalFigS31.jpg]

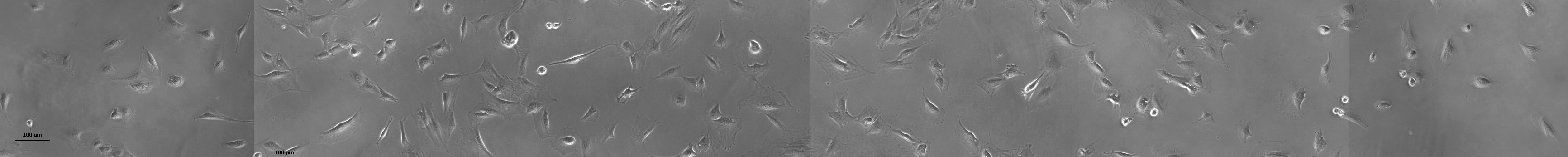

Supplement: Supplemental Information 1 — Time-lapse images of moving 3T3 fibroblast cells were captured, over a period of 12 h at 3 h intervals, using a light microscope and Eclipse TIS software at 100× magnification. Cell locations in each image were manually determined by superimposing markers onto cells and recording the Cartesian coordinates of markers using ImageJ image analysis software. [file peerj-04-1689-s003.zip › Supplemental_data/SupplementalFigS32.jpg]

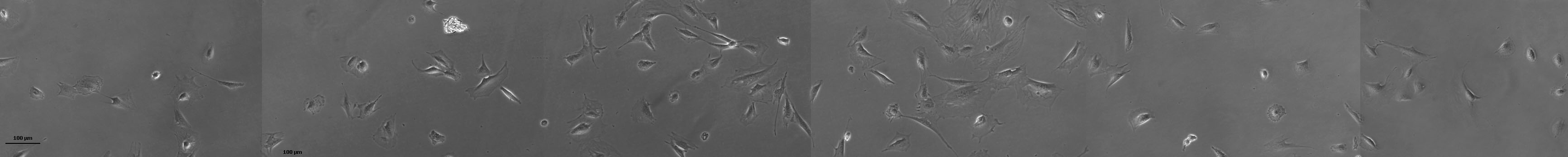

Supplement: Supplemental Information 1 — Time-lapse images of moving 3T3 fibroblast cells were captured, over a period of 12 h at 3 h intervals, using a light microscope and Eclipse TIS software at 100× magnification. Cell locations in each image were manually determined by superimposing markers onto cells and recording the Cartesian coordinates of markers using ImageJ image analysis software. [file peerj-04-1689-s003.zip › Supplemental_data/SupplementalFigS4.jpg]

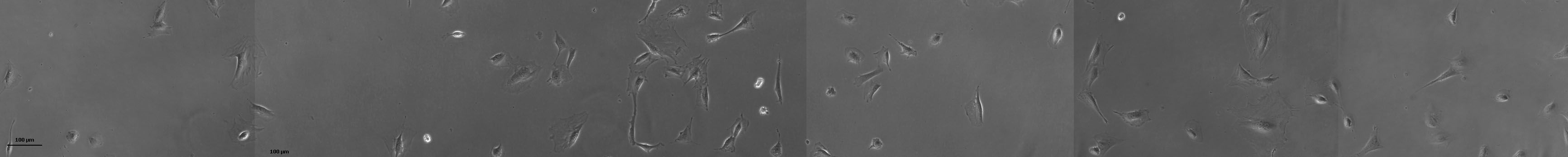

Supplement: Supplemental Information 1 — Time-lapse images of moving 3T3 fibroblast cells were captured, over a period of 12 h at 3 h intervals, using a light microscope and Eclipse TIS software at 100× magnification. Cell locations in each image were manually determined by superimposing markers onto cells and recording the Cartesian coordinates of markers using ImageJ image analysis software. [file peerj-04-1689-s003.zip › Supplemental_data/SupplementalFigS5.jpg]

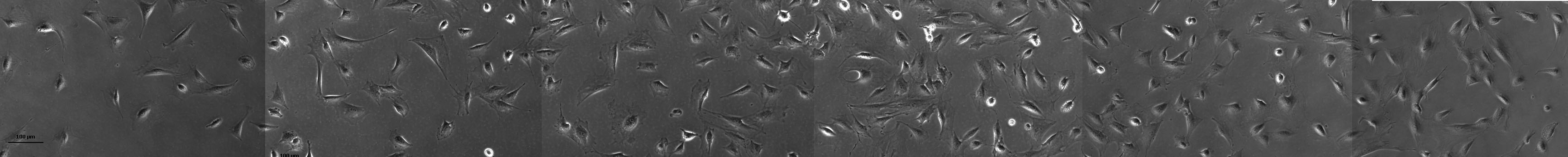

Supplement: Supplemental Information 1 — Time-lapse images of moving 3T3 fibroblast cells were captured, over a period of 12 h at 3 h intervals, using a light microscope and Eclipse TIS software at 100× magnification. Cell locations in each image were manually determined by superimposing markers onto cells and recording the Cartesian coordinates of markers using ImageJ image analysis software. [file peerj-04-1689-s003.zip › Supplemental_data/SupplementalFigS6.jpg]

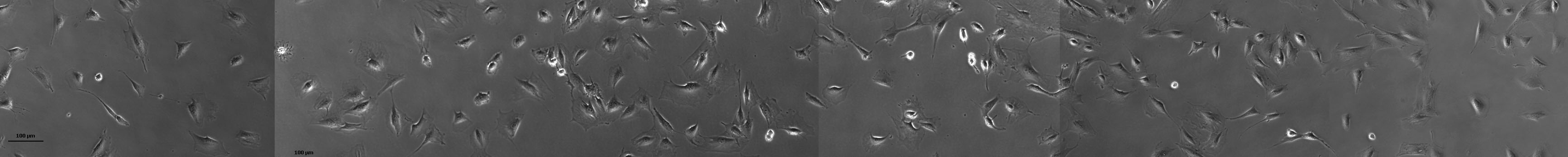

Supplement: Supplemental Information 1 — Time-lapse images of moving 3T3 fibroblast cells were captured, over a period of 12 h at 3 h intervals, using a light microscope and Eclipse TIS software at 100× magnification. Cell locations in each image were manually determined by superimposing markers onto cells and recording the Cartesian coordinates of markers using ImageJ image analysis software. [file peerj-04-1689-s003.zip › Supplemental_data/SupplementalFigS7.jpg]

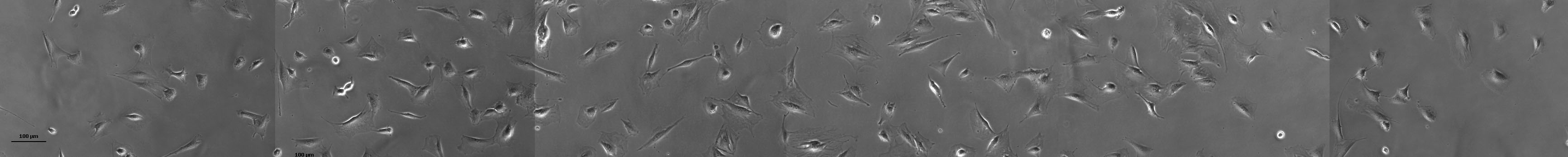

Supplement: Supplemental Information 1 — Time-lapse images of moving 3T3 fibroblast cells were captured, over a period of 12 h at 3 h intervals, using a light microscope and Eclipse TIS software at 100× magnification. Cell locations in each image were manually determined by superimposing markers onto cells and recording the Cartesian coordinates of markers using ImageJ image analysis software. [file peerj-04-1689-s003.zip › Supplemental_data/SupplementalFigS8.jpg]

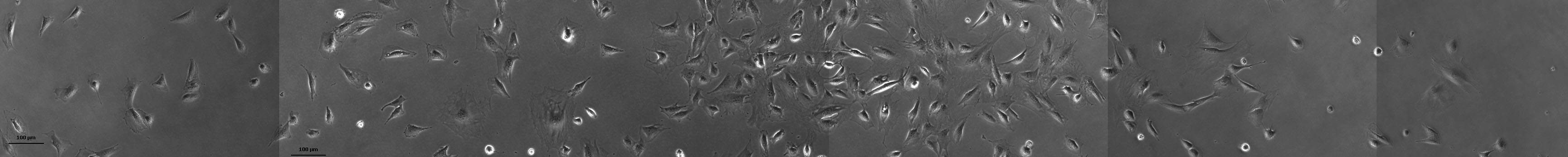

Supplement: Supplemental Information 1 — Time-lapse images of moving 3T3 fibroblast cells were captured, over a period of 12 h at 3 h intervals, using a light microscope and Eclipse TIS software at 100× magnification. Cell locations in each image were manually determined by superimposing markers onto cells and recording the Cartesian coordinates of markers using ImageJ image analysis software. [file peerj-04-1689-s003.zip › Supplemental_data/SupplementalFigS9.jpg]
